# Anticipatory gaps challenge the public governance of heritable human genome editing

**DOI:** 10.1136/jme-2023-109801

**Published:** 2024-07-02

**Authors:** Jon Rueda, Seppe Segers, Jeroen Hopster, Karolina Kudlek, Belén Liedo, Samuela Marchiori, John Danaher

**Affiliations:** 1 University of Basque Country, Leioa, Spain; 2 Department of Philosophy and Moral Sciences, Universiteit Gent, Gent, Belgium; 3 Ethics Institute, Utrecht University, Utrecht, The Netherlands; 4 Instituto de Filosfía, CSIC, Madrid, Spain; 5 Complutense University of Madrid, Madrid, Spain; 6 Delft University of Technology, Delft, The Netherlands; 7 National University of Ireland Galway, Galway, Ireland

**Keywords:** Genetic Enhancement, Genetic Engineering, Public Policy, Ethics, Morals

## Abstract

Considering public moral attitudes is a hallmark of the anticipatory governance of emerging biotechnologies, such as heritable human genome editing. However, such anticipatory governance often overlooks that future morality is open to change and that future generations may perform different moral assessments on the very biotechnologies we are trying to govern in the present. In this article, we identify an ‘anticipatory gap’ that has not been sufficiently addressed in the discussion on the public governance of heritable genome editing, namely, uncertainty about the moral visions of future generations about the emerging applications that we are currently attempting to govern now. This paper motivates the relevance of this anticipatory gap, identifying the challenges it generates and offering various recommendations so that moral uncertainty does not lead to governance paralysis with regard to human germline genome editing.

## Introduction

 Emerging technologies are ‘technologies in the making’.^1^ They are novel, fast-growing and still under research and development, but, if they mature sufficiently, they can have a prominent impact.^2 3^ The challenge is that future impacts of emerging technologies cannot be accurately predicted. Their emergence is contingent on scientific, economic, political, environmental, legal and social forces influencing technological developments. Bioethical debates about emerging biotechnologies, therefore, need to navigate many uncertainties.

Human genome editing is an example of an emerging biotechnology. Several therapeutic applications of CRISPR (Clustered Regularly Interspaced Short Palindromic Repeats) are undergoing clinical trials.^4 5^ Although we should be cautious about these investigations, somatic genome editing (ie, the genetic modification of non-reproductive cells) may become successful in the coming decades for treating a wide range of hereditary and acquired diseases, including cancer, muscle degeneration, blood, infectious, neurological, haemolytic, cardiovascular, renal, stem cell, optical, periodontitic and X-linked disorders.^6 7^ The first somatic gene therapy using CRISPR, in fact, was approved on 16 November 2023 by the UK’s Medicine & Healthcare Products Regulatory Agency and by the US Food and Drug Administration on 8 December to treat patients aged 12 and older with sickle-cell disease and β-thalassaemia.^8^,^10^ Other uses not related to somatic therapies are more uncertain. Developments in heritable genome editing are at the forefront of bioethical debates,^11^ and are, admittedly, more difficult to anticipate. Genetic enhancement—which may also be practised at the somatic level—is an application that could reshape human reproduction in the future. Technically speaking, genetic enhancement refers to ‘the use of biotechnological interventions that are not restricted to mere therapeutic goals, which aim to deliberately improve, in a beneficial way, the traits, capabilities or well-being of (generally normal or healthy) individuals by affecting their genetic endowment’ ^12^ . Still, at the reproductive level, genetic enhancement, which would hypothetically allow the improvement of non-pathological complex traits in our offspring,^13^ could take multiple decades or even centuries to become sufficiently safe and effective.

The governance of human genome editing technologies is an urgent but complicated issue. In this article, we consider an added difficulty that has not been sufficiently addressed in the ethical and social debates about emerging biotechnologies. In discussions about the public governance of biotechnologies, while it is commonly assumed that technologies are subject to change, it is seldom adequately taken into account that moral beliefs may change along with technological advances. Moreover, future generations may look differently at prospective technological possibilities that right now mostly elicit moral disapproval. We call this the ‘anticipatory gap’. By this expression, we refer to the potential (mis)match between present and future public moral views on a given technology. We argue that recognising that technologies and morality coevolve have implications for the governance of biotechnologies, motivating this issue from the discussion of public engagement with heritable human genome editing.

We focus on heritable human genome editing for various reasons. On the one hand, this topic is so popular that, following Eric Juengst, human gene editing research has already become one of the most important public debates in the history of science.^14^ On the other hand, at the academic level, human genome editing has sparked an increasing scholarship on the role of public engagement in the governance of these emerging applications.^15^,^23^ In the next section, we summarise some of the most important arguments in favour of these initiatives and mention some limitations of public engagement. That said, we believe that the contribution of this article is useful beyond the debate on heritable human genome editing. Hopefully, these reflections may be helpful for rethinking debates about the governance of other emerging technologies, although in what follows we primarily focus on heritable human genome editing.

The article is structured as follows: We begin by clarifying the meaning of ‘anticipatory governance’, highlighting the importance of public engagement in it. We also address the issue of public engagement about human genome editing and then show some limitations of these proposals. We then motivate the challenge of the anticipatory gap. We show various factors that reinforce the plausibility of significant intergenerational differences in moral perceptions of particular technological applications. After that, in an attempt to address the anticipatory gap, we offer a series of recommendations for the current governance of future (bio)technologies. We also clarify that the identification of the anticipatory gap does not lead us to a permissive or restrictive position on heritable human genome editing, nor any specific metaethical stance. Finally, we conclude by summarising our main arguments and advocating the need for more future contributions to address this underexplored challenge in bioethics.

## Public engagement for the anticipatory governance of heritable human genome editing

‘Anticipatory governance’ has been defined as ‘a broad-based capacity extended through society that can act on a variety of inputs to manage emerging (…) technologies while such management is still possible’.^24^ Public engagement has been considered an essential component of anticipatory governance.^22 24 25^ What are the main reasons for the inclusion of the ‘public’ in the governance discussion of emerging genetic technologies? At the most elementary level, public engagement can underpin the democratic legitimacy of policies on germline genome editing.^19^ This aspiration stems from the idea that society as a whole—and not just experts, institutions or markets—should decide on these emerging innovations, thus being able to collectively influence their future technological trajectories and societal impacts.^15 16^ Similarly, giving voice to the public can also be instrumentally valuable in obtaining greater epistemic plurality and not relying only on the fallible knowledge and incomplete values of experts,^26^ avoiding accordingly elitist conceptions of democracy, technocracy and epistocracy.^23^ Public participation can likewise increase society’s sense of power to influence technological development, correspondingly increasing societal trust in the advances of emerging biotechnologies.^27^

Public engagement can be understood in different ways.^17 18 22^ A typical strategy is to canvass public opinion through surveys. Indeed, public consultation (usually in the form of non-deliberative polls) on the different uses of genome editing has been notable recently. A global social media survey with respondents from 185 countries showed that therapeutic gene editing receives the majority of support while reproductive enhancement mostly generates rejection.^28^ This trend is particularly pronounced in Western countries.^20 21 29 30^ In South Africa, the moral asymmetry between therapy and enhancement in reproductive contexts is also observed, although there is significant moral support for using heritable genome editing for immunity enhancement.^15^ In some Asian countries, by contrast, public attitudes are more favourable to genetic enhancement.^31^ These consultations are valuable for measuring the public acceptance of contested technologies, and even for aligning regulatory policies with folk moral views. Furthermore, public voices can be more actively included by developing deliberative groups or citizens’ juries. The use of deliberative mini-publics on CRISPR may serve this purpose.^23^ Some have also advocated the creation of a Global Deliberative Assembly, whose objective would be to facilitate a meaningful global citizen discussion on genome editing.^32^

While there are good reasons for including public views in the anticipatory governance of genome editing, public engagement initiatives have limitations. First and foremost, social acceptance does not equate to ethical acceptability.^33^ While public consultations may provide empirical information about evaluative tendencies in society toward scientific developments, this does not in itself allow normative conclusions about how we should evaluate those developments. To infer what we collectively should do from what the majority of society currently values would be to fall into a naturalistic fallacy, namely, deriving an ethical conclusion (an ‘ought’) from a mere property of the world (an ‘is’). ^34^ Second, as mentioned by Scheufele *et al*, ‘the public’ is not a monolithic entity; instead, there are many different ‘publics’ whose values, beliefs, socioeconomic circumstances and risk perceptions are varied’.^17^ Third, public opinion (represented particularly through non-deliberative surveys) is not always the result of well-informed and careful reflection, but frequently the consequence of misinformed, biased and emotionally charged preferences.^35 36^ Fourth, and more to our point, heritable human genome editing is an evolving discussion, and public opinion may change over time.^37^ Thus, as public opinion is not stationary, the future public (whose views are uncertain) should also be considered as part of the public engagement imperative.^16^ Those limitations, among others, make public engagement a necessary but insufficient condition for anticipatory governance of heritable human genome editing.

## The anticipatory gap

Governance of emerging technologies tends to focus on social acceptance based on the norms of present morality, disregarding possible changes in future morality. In the previous section, we have argued that public engagement initiatives are undoubtedly important, although insufficient, for the governance of heritable genome editing. In what follows, we develop two more reasons to show further underexplored philosophical problems underlying these initiatives. First, some biotechnological applications—like heritable human genetic enhancement—are a long way off, if they ever become feasible. So future generations, not the present ones, will be the recipients of the benefits and harms of these technologies. What matters primarily then is the (unknowable) moral perceptions of future generations in this regard. Second, in addition to technologies, moral views may also change. Although it is impossible to predict future morality, in theory, the moral views of future generations on currently discussed emerging innovations may be different from those prevailing today. This potential (mis)match between technologies and moral attitudes between different generations is what we call the ‘anticipatory gap’. To understand the relevance and complexity of the anticipatory gap, at least four factors must be considered.

The first factor is intergenerational moral change. In recent centuries, we have witnessed the abolition of slavery, women’s emancipation, greater permissibility of same-sex behaviour and the sharp decline of practices such as honour killing, child labour or corporal punishment, to mention a few. Of course, these changes are contingent, historically specific and not universally distributed. More interestingly for this article, some moral changes are precipitated by technological and biomedical advances. These are called ‘techno-moral changes’ and are the subject of study in a growing scientific literature.^38^,^41^ The contraceptive pill prompted remarkable changes in sexual morality by separating sex from reproduction.^39 42^ The home pregnancy test increased women’s reproductive autonomy.^41^ And advances in mechanical ventilation and intensive care changed our medico-legal determination of death, together with the ethical legitimacy of organ harvesting for transplantation from ‘brain-dead’ individuals.^43^ These examples warn us that future biotechnologies may reshape our current moral beliefs and practices.

The second factor is normalisation. From a descriptive perspective, normalisation is the dynamic by which a phenomenon becomes prevalent. Normality, however, also has moral overtones. As shown by cognitive sciences, descriptive normality (the prevalence or frequency of behaviour within a population) influences prescriptive attitudes (around that same behaviour’s perceived moral correctness).^44^ Similarly, recent experimental work has shown that when enhancements are perceived as normal (ie, as frequent practices), moral opposition to them decreases.^45^ Hence, normalisation could affect public views on germline genome editing in the future. Indeed, in the past, other procedures in reproductive medicine that initially provoked rejection ended up being more broadly accepted as normal or less morally problematic, such as amniocentesis, heterologous artificial insemination, in vitro fertilization or preimplantation genetic diagnosis.^46 47^ Familiarity with genetic science, moreover, already seems to favourably modulate attitudes in support of genome editing.^20 22^ Therefore, it is plausible that the perception of normality may modulate the (de)moralisation of certain practices linked to the use or avoidance of emerging biotechnologies.

A third factor is the coexistence of different moral perspectives. The surveys mentioned above show the disparity in attitudes towards the different uses of genome editing. There may indeed be majority views for or against specific uses. But, again, what the majority thinks does not necessarily determine what should be allowed or prohibited. In any case, public morality is characterised by plurality. This divergence of moral views can influence technology adoption, both at the international and intrastate levels. On the one hand, variations in public perceptions between countries, along with the defence of national sovereignty over science and technology legislation, may lead to significant differences in the adoption of heritable human gene editing globally. ^48^ On the other hand, paying attention to the values of early adopters is critical. In order to identify possible ways of social change, it is essential to bear in mind that ‘first movers’ can have trendsetting effects.^49^ Hence, pioneer users of biotechnologies set patterns that can be followed by others, changing the mainstream trends.

A last factor is that humans have a status quo bias.^50^ The tendency to favour the status quo means that we have an inclination to oppose change. This bias may have affected the human gene editing debate.^51^ This tendency can also sometimes explain why we are reluctant to encourage certain technological changes. Moreover, this cognitive limitation, coupled with our temporal parochialism, may make it more difficult to imagine that there could be changes between present and future morality. Favouritism of our technomoral status quo can be among many other cognitive factors that lead to motivated reasoning that reinforces people’s initial moral views.

All in all, these four factors show us the complexity of the anticipatory gap. The first three factors support the idea that intergenerational moral changes, which are sometimes mediated by technological developments, value pluralism and cognitive normalisation processes, are not infrequent phenomena. The last factor shows, by contrast, that a preference for the current technomoral status quo may prevent proper consideration of future generations’ preferences regarding heritable human gene editing. Needless to say, this is not an exhaustive analysis, and other factors—requiring further investigation—can certainly play a role in this phenomenon.

## Bridging the gap

How should we govern future technologies if we do not know what future generations, who will be affected by their uses, will think of them? How should we bridge the anticipatory gap? In this section, we offer recommendations to avoid governance paralysis.

It is important to note that, in this article, we remain agnostic about the moral permissibility of heritable human genome editing, either for therapeutic or enhancement purposes. The anticipatory gap does not imply a permissive stance on the prospective employment of emerging technologies. Our point here is that the ethical justifications for (not) allowing such implementations may fluctuate as we see the actual development of these technologies. Scientific advances will shape the degree of safety, effectiveness and accessibility of these technologies, which may probably affect expert ethical analyses and lay moral perceptions.

The anticipatory gap, likewise, does not necessarily entail a kind of intergenerational moral relativism. As a stance on the truth value of moral beliefs, moral relativism asserts that right and wrong vary from culture to culture, or, in this case, from time to time. We do not claim that our—diverse and perhaps contradictory—current moral views on germline gene editing are wrong, nor that future generations will be right, or vice versa. We only claim that moral views on biotechnologies, and their justifications, are malleable by sociocultural factors and technological evolution itself. In any case, it should be noted that there is not an abysmal difference in values between generations. In fact, some values (such as freedom, safety, benevolence or fairness) have remained prominent throughout human history, even between non-overlapping generations.^52^ It is possible—and even desirable—that these remarkably prevalent moral ideals remain influential in the future. Furthermore, since we should not exclude that the adoption of heritable human genome editing might not be that far off, value overlap between generations is obviously possible.

Given these two clarifications, we now present four recommendations.

First, a consequence of the anticipatory gap is that the governance of emerging biotechnologies must be iterative. That is, anticipatory governance has to be updated periodically. In the face of the possibility of future technomoral changes, at the theoretical level, we advocate the need for epistemic humility, open-mindedness and adaptability on the part of governing institutions.^53^ This does not mean that we should resist positively influencing future generations (and even their values). Rather, it is just that ethical evaluation cannot be once-and-for-all but must be revisable and dynamic. At the practical policymaking level, different measures can be envisioned. As provisionality is a characteristic of regulatory efforts on human gene editing,^54^ using legislation models with ‘sunset clauses’ can be a fruitful example to impede temporary moratoriums from becoming effective bans resistant to change.^55^ Moreover, as has been proposed in the governance of intergenerational climate risks, adaptive planning is a useful strategy for readjusting policies according to how the future unfolds.^56^ Renewing the planning should be part of the plan when dealing with evolving biotechnologies.

Second, it is advisable to build a societal response capacity, based on the conjunction between expert support and public participation,^57^ which is flexible and adaptable to different scenarios.^58^ The future is not univocal. There may be multiple technomoral scenarios, as our present actions may bifurcate us to different futures. This vision is certainly a rejection of technological determinism: we are not passively destined for a particular technological future. It is necessary, therefore, to strengthen the tasks of foresight in order to be prepared for various plausible futures,^22^ in which technologies and moral values interact differently.^59^ In addition to expert foresight bodies, promoting public engagement can be a way to make this social preparation for the various biotechnological futures more participatory and democratic. That said, it is important to compare the standards of the public with those of the experts regarding the approval or rejection of the various scenarios.

A third recommendation is to pay attention to how moral evaluations may change according to the particularities of different populations. Biotechnological developments do not affect everyone equally. Although many people might not have a defined moral appraisal of the future uses of heritable human genome editing today, this may change as it becomes clearer how technological advances may affect the collectives to which they belong. Accordingly, an interesting strategy, which has also been proposed in the climate change debate, is to use methods of representation of groups that may be more adversely affected in the future.^60^ Representing the interests of future collectives is undoubtedly difficult, but it should be considered in discussions on heritable human genome editing.

Finally, we must consider the long-lasting impacts of biotechnologies that may become entrenched in society. One risk is that we get stuck with technologies that we no longer value in the same way. The ‘technological lock-in’ problem explains why some technologies that modify our social structures become resistant to change, which becomes problematic if our values evolve. Because of the phenomenon of increasing returns, the more a technology is socially adopted, the more it tends to be innovated to improve its performance, which in turn increases its social adoption.^61^ Gas-powered cars are examples of ‘technological lock-in’ since they have become persistent and costly to renounce, even if the value of sustainability pushes us to see them as more morally problematic. Applied to the case of heritable human genome editing, this phenomenon forces us to glimpse the long term, enduring impacts of these technologies on society. For instance, if germline genome editing becomes truly effective, omitting its use may become costly. This could increase its adoption and make us more dependent on this biotechnology, which could have unanticipated effects on other values, such as increasing the stigma of those who forgo its use. While it is impossible to know the future trade-off between values, it is plausible that future generations may regret some innovations that become stagnant, all things considered. Therefore, the discussion on the possible lasting consequences of genetic technologies on the future of humanity must be deepened.

## Concluding remarks

The anticipatory gap compels us to rethink the governance of biotechnologies, bearing in mind that technologies and morality coevolve. Today’s moral views on emerging biotechnologies are not immutable. Future generations may not only have different innovations but also different moral beliefs on these very technologies. Importantly, uncertainty about future technomoral visions should remind us that public engagement initiatives, while essential, are not sufficient on their own to resolve the anticipatory governance of emerging technologies, especially those with more remote effects on future generations.

Although, in this article, we have motivated this problem from the pressing case of heritable human genome editing, what we have said here can apply to other emerging biotechnologies. We believe, moreover, that this issue is underdeveloped in the bioethics literature and requires further academic attention in the future. We hope, therefore, that this article will encourage further contributions that discuss how the interests of future generations should be represented in debates about the impacts of emerging disruptive biotechnologies.

Finally, anticipatory governance initiatives should carefully consider the evolving moral perceptions of biotechnologies. Uncertainty, of course, will be an inevitable travel companion. As the traveller who moves through uncharted waters, the anticipatory governance of emerging technologies operates in a terra incognita. Yet, what is fairly certain is that throughout this journey, perhaps fraught with technomoral turbulence, public ethical discussion will continue to be essential in the future.

## Data Availability

No data are available.
